# Phosphorylation-Dependent Ubiquitination of Paraxial Protocadherin (PAPC) Controls Gastrulation Cell Movements

**DOI:** 10.1371/journal.pone.0115111

**Published:** 2015-01-12

**Authors:** Masatake Kai, Naoto Ueno, Noriyuki Kinoshita

**Affiliations:** 1 Department of Developmental Biology, National Institute for Basic Biology, 38 Nishigonaka, Myodaiji-cho, Okazaki, Aichi 444-8585, Japan; 2 Department of Molecular Biomechanics, SOKENDAI (The Graduate University for Advanced Studies), 38 Nishigonaka, Myodaiji-cho, Okazaki, Aichi 444-8585, Japan; Seoul National University, KOREA, REPUBLIC OF

## Abstract

Paraxial protocadherin (PAPC) has been shown to be involved in gastrulation cell movements during early embryogenesis. It is first expressed in the dorsal marginal zone at the early gastrula stage and subsequently restricted to the paraxial mesoderm in *Xenopus* and zebrafish. Using *Xenopus* embryos, we found that PAPC is also regulated at the protein level and is degraded and excluded from the plasma membrane in the axial mesoderm by the late gastrula stage. Regulation of PAPC requires poly-ubiquitination that is dependent on phosphorylation. PAPC is phosphorylated by GKS3 in the evolutionarily conserved cytoplasmic domain, and this in turn is necessary for poly-ubiquitination by an E3 ubiquitin ligase β-TrCP. We also show that precise control of PAPC by phosphorylation/ubiquitination is essential for normal *Xenopus* gastrulation cell movements. Taken together, our findings unveil a novel mechanism of regulation of a cell adhesion protein and show that this system plays a crucial role in vertebrate embryogenesis.

## Introduction

Co-ordinated cell migration is fundamental to various biological processes including morphogenesis, wound healing and cancer metastasis. In gastrulating *Xenopus* embryos, for example, lateral and chordal mesodermal cells are polarized and aligned mediolaterally, and simultaneously migrate towards the midline and intercalate each other to narrow and elongate the body axis, a process known as convergent extension (CE) [1]. Here, cells are maintained as a cohesive tissue while individual cells break the cell-cell boundary by wedging between neighbouring cells, therefore it is proposed that regulation of cell adhesion/deadhesion is a prerequisite for such cell behaviour [2].

Paraxial protocadherin (PAPC) belongs to the protocadherin family, and has six cadherin ectodomains, a single-pass transmembrane domain and a cytoplasmic tail [3]. Prior to the onset of gastrulation, PAPC is expressed in the Spemann organizer in *Xenopus* and the dorsal side of the marginal zone in zebrafish [3,4]. During gastrulation, its expression is suppressed in the axial mesoderm and restricted to the paraxial mesoderm [3,4]. PAPC has been shown to play roles in several aspects of *Xenopus* embryogenesis, including CE [3,5,6] and tissue separation [7–9]. Nevertheless, PAPC has been demonstrated to exert its biological function by modifying cell adhesion property, at least in part through C-cadherin [9,10], as well as acting as a signalling molecule to regulate downstream factors such as RhoA, JNK [5,8] and Sprouty [6,11,12].

PAPC localization is dynamically controlled during *Xenopus* gastrulation. One important mechanism to regulate PAPC localization and stability is the Wnt/planar cell polarity (PCP) pathway [10]. Wnt/PCP signalling stabilizes PAPC on the plasma membrane, whereas loss of Wnt/PCP signalling promotes PAPC internalization and clustering in the cytoplasm in *Xenopus* embryonic cells [10]. However, the precise molecular mechanism by which PAPC localization is regulated is not fully understood.

Plasma membrane localization and internalization of many membrane proteins are regulated by mono- and poly-ubiquitination. Ubiquitination of the client protein, catalyzed by the E3 ubiquitin ligase complex, serves as a signal for a variety of cellular processes, such as protein degradation, localization and activation [13]. This variation of cellular roles for ubiquitination derives from the different linkages of ubiquitin polymerization. For example, lysine-48–linked ubiquitin chains promote proteasomal degradation of the target proteins. Lysine-63–linked ubiquitin chains on membrane proteins lead to their endocytosis and down-regulation [14].

In this study, we show that phosphorylation of PAPC by GSK3 and subsequent poly-ubiquitination by β-TrCP is crucial for regulation of PAPC localization and stability. At an early gastrula stage, PAPC is recycled to the plasma membrane, while by the early neurula stage PAPC is degraded and excluded from the plasma membrane in the axial mesoderm. Disruption of this system in *Xenopus* early embryos leads to abnormal morphogenesis typical of gastrulation defects. Thus, our findings suggest a novel mechanism of regulation of a cell adhesion protein, a system that plays a crucial role in vertebrate embryogenesis.

## Results

### PAPC is down-regulated in the axial mesoderm cells undergoing convergent extension

**Figure 1:**
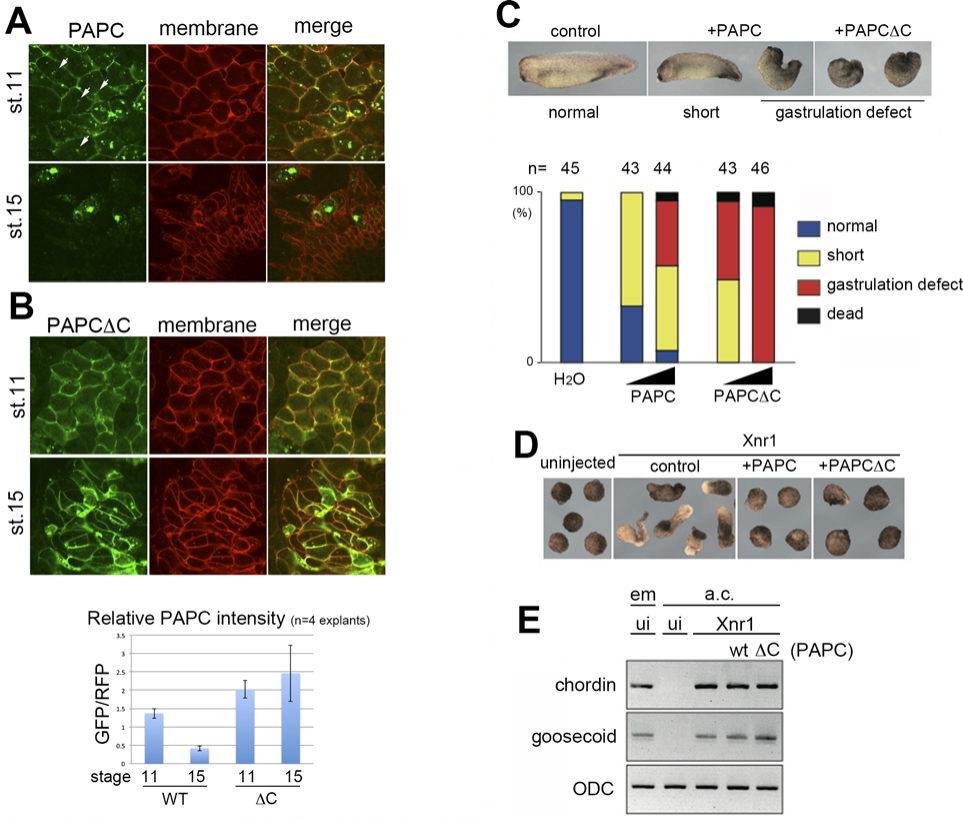
PAPC is down-regulated in the dorsal marginal zone explants at late gastrula. (**A**) GFP-tagged PAPC (PAPC-GFP) was expressed in the dorsal marginal zone (DMZ) of a *Xenopus* embryo. In the DMZ explant at stage 11 (early gastrula), PAPC-GFP was found mainly on the plasma membrane (marked by membrane-targeted RFP), with some cytoplasmic puncta (arrowheads). At stage 15 (early neurula), PAPC-GFP signal was greatly reduced and excluded from the plasma membrane. (**B**) GFP-tagged PAPC mutant lacking the cytoplasmic domain (PAPCΔC) was not degraded and persistently localized to the plasma membrane at stages 11 and 15. PAPC-GFP signal was reduced at stage 15 as measured by relative fluorescent intensity using membrane-RFP as an internal control, while PAPCΔC-GFP signal persisted. (**C**) Injecting an increasing amount of wild-type PAPC mRNA into *Xenopus* embryos induced more severe gastrulation defects, including a short body axis and spina bifida at stage 28. PAPCΔC over-expression exhibited stronger effects. The amount of injected RNAs was 200 and 1000 pg, respectively. (**D**) Over-expression of PAPC or PAPCΔC inhibited elongation of animal caps mesodermalized by Xnr1 expression. (**E**) Induction of mesoderm was confirmed by expression of *chordin* and *goosecoid* (*gsc*) by RT-PCR. em, embryos; a.c., animal caps; ui, uninjected.

To investigate the properties of PAPC in *Xenopus* embryos, we expressed GFP-tagged PAPC (PAPC-GFP) in the dorsal marginal zone (DMZ) and observed the explants at stages 11 and 15 (Fig. 1A). At stage 11, PAPC-GFP localized primarily to the plasma membrane (Fig. 1A). We also noticed some vesicular localization of PAPC in the cytoplasm (Fig. 1A, arrowheads). At stage 15, the PAPC-GFP signal was greatly reduced, especially on the plasma membrane from where it was mostly excluded (Fig. 1A). Regulation of PAPC is mediated through its C-terminal cytoplasmic domain, as PAPC lacking this domain (PAPCΔC) was not degraded and persistently localized to the plasma membrane throughout stages 11 to 15 in the DMZ explant (Fig. 1B). Measuring the signal intensity against the internal control (membrane-RFP) confirmed PAPC-GFP degradation and PAPCΔC-GFP persistence at stage 15 (Fig. 1B graph).

Knock-down of PAPC by antisense morpholino oligonucleotides (MO) in *Xenopus* embryos is known to induce defects in gastrulation cell movements such as a short body axis and spina bifida in whole embryos and inhibition of elongation of mesodermalized animal caps [5]. Given its expression patterns, these studies indicated that PAPC is required cell-autonomously in the dorsal marginal zone before the onset of gastrulation and/or non-cell-autonomously in the axial mesoderm during gastrulation to facilitate CE (see discussion). We tested whether suppression of PAPC is also required for normal gastrulation by over-expressing PAPC in *Xenopus* embryos. Here we found that injecting an increasing amount of PAPC mRNA led to more severe gastrulation defects in whole embryos (Fig. 1C). A cell movement defect by PAPC over-expression was also observed in mesodermalized animal caps (Fig. 1D). Expression of PAPCΔC, which showed sustained membrane localization, led to even more severe cell movement defects compared to wild-type PAPC both in whole embryos (Fig. 1C) and mesodermalized animal caps (Fig. 1D and E).

We next sought to discriminate PAPC stability in the axial and paraxial tissues. To circumvent the adverse effects of expressing full-length PAPC, we constructed EGFP-PAPCΔE, in which the ectodomain of PAPC is replaced with EGFP (Panel A in S1 Fig.). In the DMZ explants, EGFP-PAPCΔE signal in the notochordal region was markedly lower than that in the neighbouring tissue at stage 17 (Panel B in S1 Fig.). Observing the embryo in cross-section at stage 17 further revealed the exclusion of EGFP-PAPCΔE in the notochord (Panel C in S1 Fig.), suggesting that PAPC is controlled not only at the mRNA level but also at the protein level in the axial mesoderm.

Taken together, these results argue that, on top of its required role, PAPC has to be down-regulated in the axial mesoderm for normal cell movement to occur during gastrulation, both at the mRNA and protein levels.

### The evolutionally-conserved region in the cytoplasmic domain is required for normal membrane localization of PAPC

**Figure 2:**
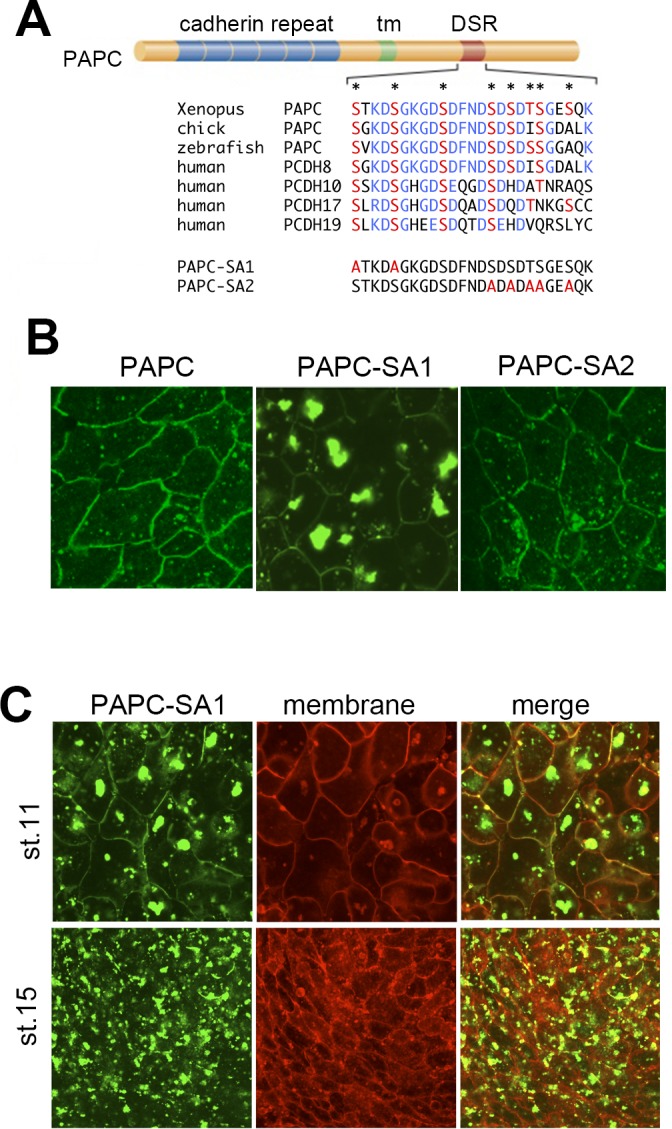
Phosphorylation of PAPC by GSK3 at the DSR domain is required for PAPC regulation. (**A**) Evolutionarily conserved domain of PAPC in the cytoplasmic region. This domain is rich in aspartic acid and serine and is referred to as the DSR domain. The sequence of mutants used in this study (SA1, SA2) is also indicated. (**B**) PAPC; wild-type PAPC tagged with GFP, PAPC-SA1; a putative non-phosphorylated mutant in which S816 and S820 are replaced with alanine, PAPC-SA2; S830, S832, T834, S835 and S838 are replaced with alanine. mRNAs encoding these proteins were injected into the animal pole of *Xenopus laevis* embryos. Animal caps were dissected and observed under a microscope. (**C**) PAPC-SA1 mRNAs were injected into the dorsal marginal zone. The mutant was maintained in the DMZ explant at stage 15 when wild-type PAPC is mostly degraded.

Apart from the cadherin repeats in the extracellular domain and the transmembrane domain, PAPC has an evolutionarily conserved domain in the cytoplasmic region [15] (Fig. 2A). As this ∼25-amino acid stretch is rich in aspartic acid (D) and serine (S), we will refer to this region as the DSR (D- and S-rich) domain. Since the DSR domain in *Xenopus* has a total of nine serine/threonine residues, we postulated that phosphorylation might play a role in its function. We therefore generated a series of ‘non-phosphorylated’ PAPC mutants by mutating these residues and tested their subcellular localization. Changing five serine/threonine residues on the C-terminal side (S830, S832, T834, S835 and S838) to alanine (PAPC-SA2-GFP; Fig. 2A) had little effect on PAPC localization (Fig. 2B) compared with the wild type. By contrast, a PAPC mutant in which two serines on the N-terminal side (S816 and S820) in the DSR domain were replaced with alanine (PAPC-SA1-GFP; Fig. 2A) showed reduced localization to the plasma membrane and formed large cytoplasmic aggregates in animal cap cells (Fig. 2B), indicating that these serine residues in the DSR domain are crucial for the regulation of PAPC localization. Cytoplasmic aggregates of PAPC-SA1-GFP, accompanied with reduced plasma membrane localization, were also observed in DMZ explants at stage 11 (Fig. 2C). PAPC-SA1-GFP localized to the cytoplasmic vesicles in a dose-independent manner, since injecting lower amount of PAPC-SA1-GFP mRNA (100 pg/embryo, as compared to 500 pg in other experiments) also resulted in GFP-positive vesicle formation (S2 Fig.). At stage 15, when wild-type PAPC is largely degraded, the PAPC-SA1-GFP signal was markedly retained, mostly in the cytoplasm.. We therefore conclude that these serine residues are important for PAPC localization and stability in *Xenopus* early embryogenesis.

**Figure 3:**
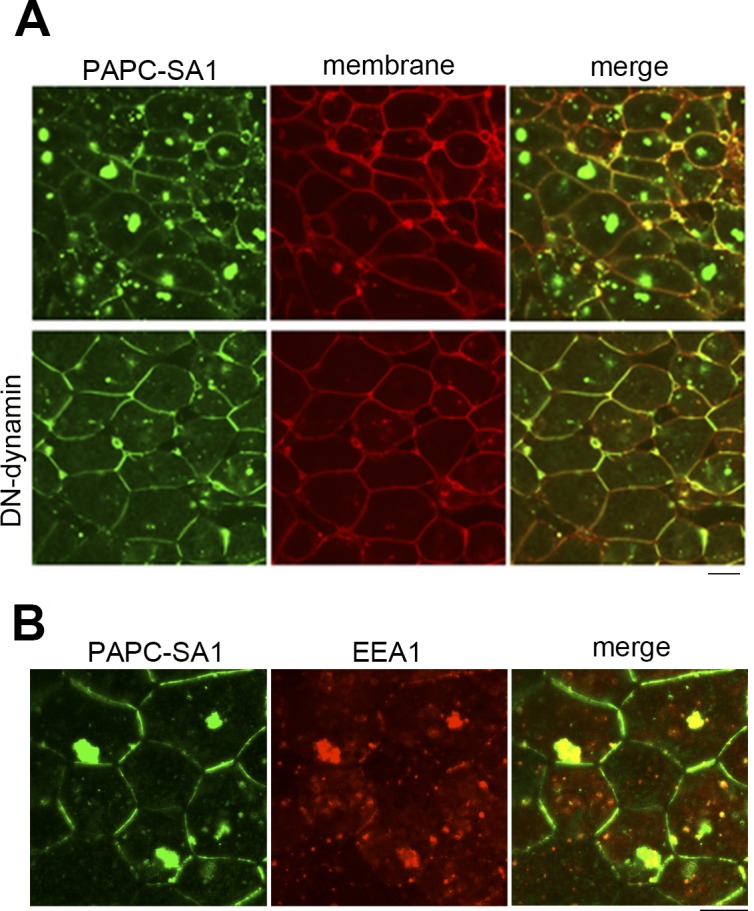
PAPC localization is regulated by endocytic recycling. (**A**) PAPC-SA1 and membrane-RFP were co-expressed in animal cap cells (upper panel). PAPC-SA1, membrane-RFP and dominant-negative (DN) dynamin were co-expressed (lower panel). (**B**) PAPC-SA1 was co-expressed with Early endosome antigen-1 (EEA1) tagged with RFP.

The cytoplasmic accumulation of PAPC-SA1-GFP may be due to the inhibition of a secretory pathway or the inhibition of endosomal recycling. In order to clarify how the aggregation formed, the dominant-negative dynamin mutant (DN-dynamin), which inhibits endocytosis, was co-expressed with PAPC-SA1. As shown in Fig. 3A, DN-dynamin drastically reduced the aggregation of PAPC-SA1. In addition, these aggregates are colocalized with the early endosomal marker EEA1 (Fig. 3B). These results support the possibility that the mutation in the DSR domain of PAPC impairs the normal endosomal recycling system.

### GSK-3 phosphorylates PAPC at the DSR domain

**Figure 4:**
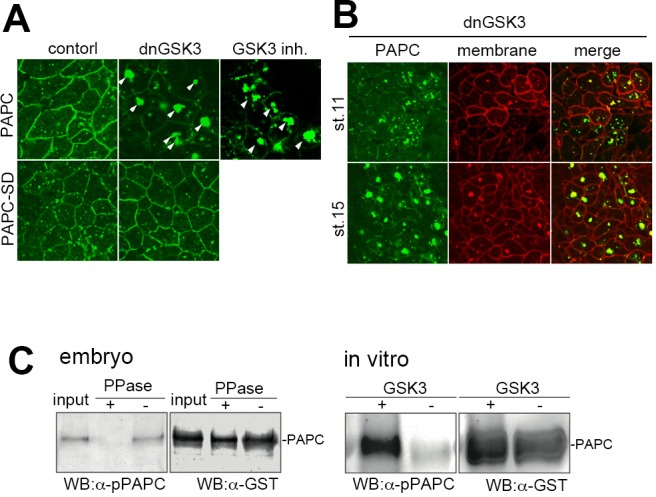
GSK3 is involved in the regulation of PAPC localization and stability. (**A**) Inhibiting GSK3 activity by expression of dominant-negative GSK3 (dnGSK3), or treating explants with a GSK3 inhibitor BIO one hour prior to observation, led to extensive aggregation of PAPC in the cytoplasm in animal cap cells (arrowheads). A putative phosphorylation-mimic mutant PAPC-SD, which carries S816 and S820 to aspartic acid mutations, was resistant to dnGSK3 expression and remained on the plasma membrane. (**B**) PAPC was not efficiently degraded in DMZ explants expressed with dominant-negative GSK3 (dnGSK3) at stage 15 and formed cytoplasmic aggregates. (**C**) GST-tagged PAPC was expressed in the dorsal marginal zone. When embryos reached the gastrula stage, PAPC-GST was partially purified with Glutathione Sepharose 4B. Phosphorylated PAPC (pPAPC) was detected by anti-phosopho-PAPC antibody, raised against phosphorylated DSR peptide as an antigen. The pPAPC signal was abolished by treating the extract with lambda protein phosphatase (PPase). The recombinant C-terminal domain of PAPC was phosphorylated in vitro in the presence of recombinant GSK3, as confirmed by a Western blot using anti-pPAPC antibody. Total input of PAPC was estimated by anti-GST antibody.

Because S816 and S820 in the DSR domain of *Xenopus* PAPC conform to the GSK3 consensus motif (Ser-X-X-X-Ser[p]), where downstream D824 may serve as a constitutive priming residue,, we next investigated whether GSK3 is involved in the regulation of PAPC. Expression of a dominant-negative GSK3 (dnGSK3) [16] reduced the PAPC-GFP signal on the plasma membrane (Fig. 4A, upper right panel), and induced the formation of large aggregates in the cytoplasm (arrowheads in Fig. 4A, upper middle panel), in a similar manner to PAPC-SA1-GFP. As expressing dnGSK3 from the earliest stages of development by mRNA injection is expected to cause pleiotropic effects, we treated the explants with a GSK3 inhibitor BIO one hour prior to the observation, and confirmed it too led to PAPC-GFP aggregates formation (Fig. 4A, upper right panel). On the other hand, a ‘phosphorylation-mimic’ PAPC mutant, in which S816 and S820 are replaced with aspartic acid (PAPC-SD; Fig. 2A), was resistant to dnGSK3 expression and whose wild-type-like localization was not altered (Fig. 4A, lower right panel). Degradation of PAPC in DMZ explants at stage 15 was also blocked by dnGSK3 expression (Fig. 4B). By using an antibody specifically raised to recognize phosphorylation at these residues (anti-pPAPC Ab), we confirmed that PAPC is indeed phosphorylated in *Xenopus* embryos at stage 11 (Fig. 4C). In vitro analysis further revealed that purified GSK3 is able to phosphorylate the cytoplasmic domain of PAPC expressed in *E. coli* (Fig. 4C). These results strongly support the argument that phosphorylation at S816 and/or S820 by GSK3 is essential for normal PAPC regulation during *Xenopus* gastrulation.

### PAPC poly-ubiquitination facilitates PAPC localization to the plasma membrane

**Figure 5:**
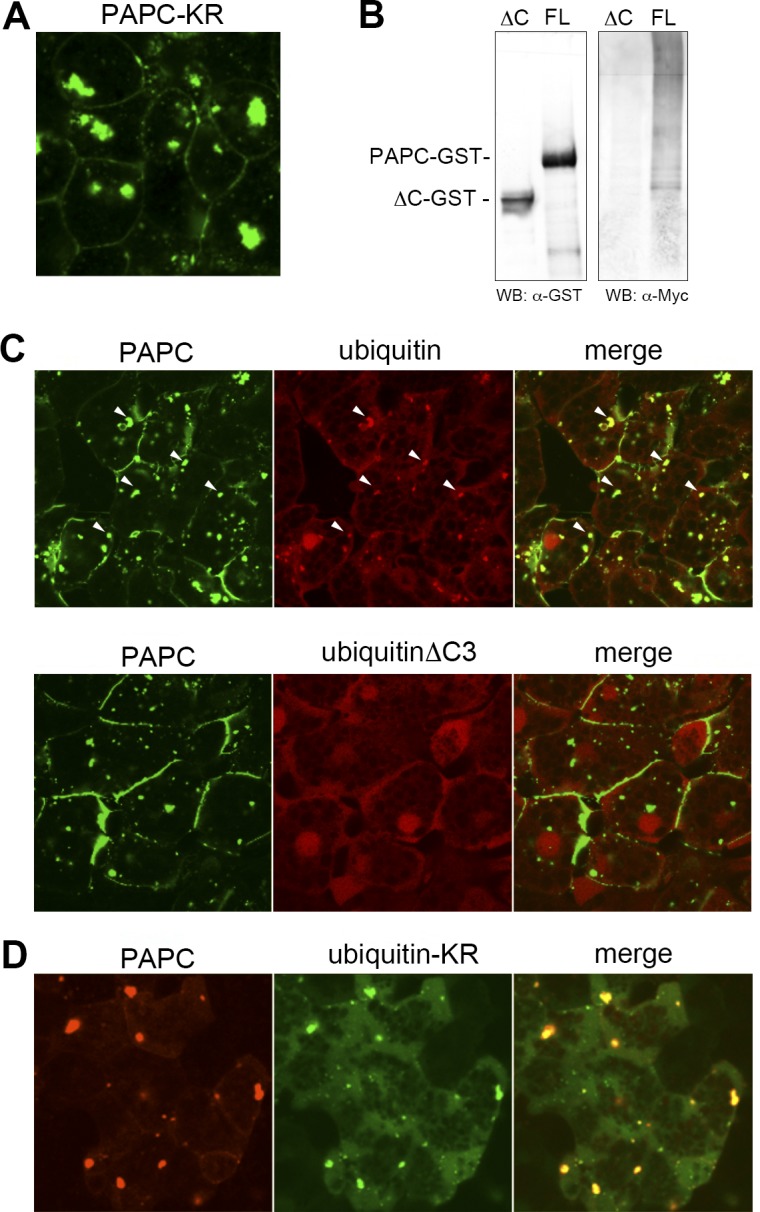
Poly-ubiquitination in the cytoplasmic vesicle regulates PAPC localization. (**A**) Changing nine lysine residues in the cytoplasmic region of PAPC to arginine resulted in PAPC aggregation in the cytoplasm, indicating the role of ubiquitination in PAPC localization. (**B**) The ubiquitination assay revealed that PAPC is poly-ubiquitinated in the *Xenopus* embryo. Myc-tagged ubiquitin co-expressed with GST-tagged full-length PAPC (FL) in the *Xenopus* embryo was purified with PAPC-GST and detected as a high molecular weight ladder. Deletion of PAPC cytoplasmic domain (ΔC) diminished poly-ubiquitination. (**C**) RFP-tagged ubiquitin and GFP-tagged PAPC co-localized in the cytoplasmic vesicle in animal cap cells (arrowheads). By contrast, RFP-tagged UbiquitinΔC3, which has three amino acids at the C-terminus removed and therefore cannot be conjugated to the target, did not co-localize with PAPC-GFP in animal cap cells. GFP-tagged ubiquitin-KR, which is conjugated to the target but blocks formation of poly-ubiquitin, co-localized with PAPC-RFP in the cytoplasmic vesicles. Ubiquitin-KR-GFP-expressing cells from which PAPC had been depleted from the plasma membrane exhibited a round morphology indicative of reduced cell-cell adhesion.

By analogy to regulation of β-catenin, which is ubiquitinated by β-TrCP subsequent to phosphorylation by GSK3 [17–19], we were curious to determine whether ubiquitination is involved in PAPC regulation. First, we tested a ‘non-ubiquitinated’ PAPC mutant that has nine lysine residues within the cytoplasmic region changed to arginine (PAPC-KR), and found that it also forms cytoplasmic aggregates in the animal cap cells (Fig. 5A), suggesting the role of ubiquitination in PAPC regulation. We then assessed whether PAPC is actually ubiquitinated, by expressing Myc-tagged ubiquitin (Myc-Ubi) together with PAPC-GST, followed by purification of PAPC with GST and the detection of conjugated ubiquitin by an anti-Myc Western blot. Here we found that PAPC is indeed poly-ubiquitinated in *Xenopus* embryos, as Myc-Ubi was detected as a high molecular weight ladder (Fig. 5B). Myc-Ubi ladder was detected with highly-purified PAPC in a denaturing condition (6M urea, S3 Fig.), strongly suggesting that poly-ubiquitin chain was directly and covalently bound to PAPC. PAPCΔC showed markedly reduced ubiquitination relative to the wild type (Fig. 5B and S3 Fig.). When PAPC-GFP was co-expressed with RFP-tagged ubiquitin in animal cap cells, their co-localization was observed in the cytoplasmic puncta (Fig. 5C). UbiquitinΔC3, in which three C-terminal amino acids that are expected to be essential for ubiquitin conjugation to the substrate are removed, did not co-localize with PAPC-GFP (Fig. 5C). These results suggest that the poly-ubiquitin chain is conjugated mainly to the cytoplasmic domain of PAPC in the cytoplasmic vesicles.

The ubiquitin mutant that has a lysine-to-arginine substitution (Ubi-KR) successfully conjugated to the substrate but failed to form a poly-ubiquitin chain. We assessed the role of PAPC poly-ubiquitination by expressing GFP-tagged Ubi-KR (GFP-Ubi-KR). As expected, GFP-Ubi-KR co-localized with PAPC-RFP in the cytoplasmic vesicles (Fig. 5D). Simultaneously, the PAPC signal was greatly reduced on the plasma membrane. Ubi-KR-expressing cells appeared to be rounder (Fig. 5D), implicating reduced cell adhesion. Taken together, we suggest that poly-ubiquitination of PAPC facilitates PAPC localization to the plasma membrane and cell-cell adhesion in *Xenopus* early embryos.

### β-TrCP interacts with and ubiquitinates PAPC

**Figure 6:**
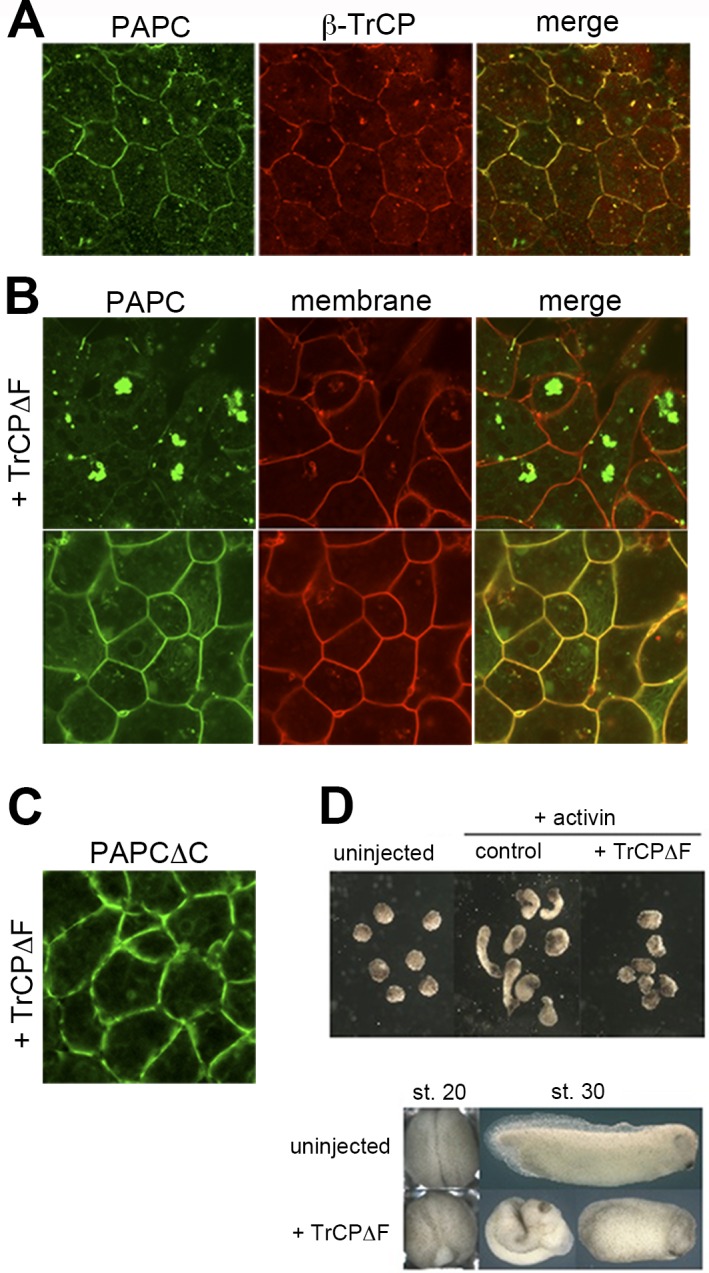
β-TrCP interacts with and ubiquitinates PAPC and is required for normal *Xenopus* embryogenesis. (**A**) β-TrCP tagged with RFP co-localized with PAPC-GFP in animal cap cells, both on the plasma membrane and cytoplasmic punta (arrowheads). (**B**) Expression of TrCPΔF, a dominant-negative form of TrCP that lacks the F-box essential for E3 ligase complex formation, induced PAPC cytoplasmic aggregates in animal cap cells. Note the cells that appear rounder than those in (A). (**C**) GFP-tagged PAPCΔC was co-expressed with TrCPΔF in *Xenopus* DMZ cells. (**D**) TrCPΔF expression inhibited animal cap elongation when treated with activin. TrCPΔF also gave rise to gastrulation phenotypes such as defective blastopore closure and a shortened body axis.

We next sought the ubiquitin ligase responsible for PAPC ubiquitination. We found that RFP-tagged β-TrCP, an E3 ligase involved in β-catenin regulation [17], co-localizes with PAPC-GFP on the plasma membrane as well as in small cytoplasmic vesicles in animal cap cells (Fig. 6A). Co-expression of PAPC-GFP and TrCPΔF, a dominant negative form of β-TrCP that lacks the domain (F-box) essential for E3 ligase complex formation [18], resulted in PAPC cytoplasmic aggregation and exclusion from the plasma membrane in animal cap cells (Fig. 6B), further emphasizing the role of ubiquitination by β-TrCP in PAPC regulation. Reminiscent of Ubi-KR-expressing cells, TrCPΔF-expressing cells showed a rounder morphology (Fig. 6B) in the animal cap, again implying the role of PAPC regulation in cell adhesion. PAPCΔC, which shows persistent localization to the plasma membrane, was not affected by TrCPΔF expression in the animal cap (Fig. 6D). Correspondingly, blocking β-TrCP function by expression of TrCPΔF also inhibited elongation of mesodermalized animal caps (Fig. 6E), and delayed yolk plug closure at stage 20 and caused the body axis to be shortened at stage 28 in whole embryos (Fig. 6E). These data show that β-TrCP is required for proper PAPC localization and efficient cell movements in *Xenopus* early development.

**Figure 7:**
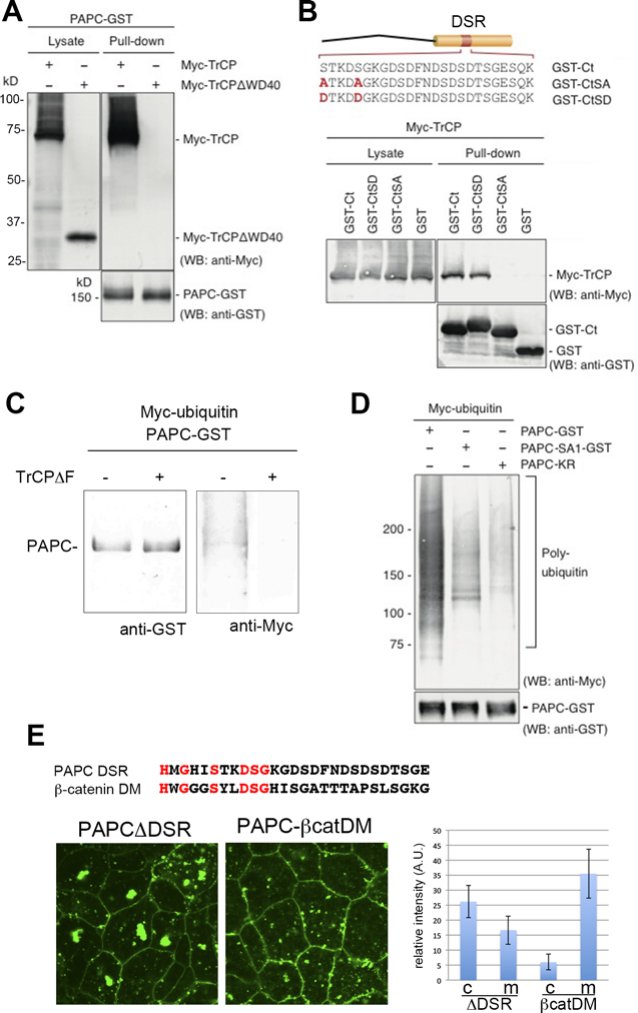
Phosphorylation-dependent β-TrCP interaction and ubiquitination of PAPC. (**A**) GST pull-down assay between PAPC-GST and β-TrCP. β-TrCP was pulled-down by PAPC, whereas PAPC without the substrate recognition domain (WD40 repeat; β-TrCPΔWD40) did not display an interaction with PAPC. Western blot bands of Myc-β-TrCP have smears in the upper regions. These are due to poly-ubiquitination of β-TrCP. (**B**) C-terminal domains of wild-type (Ct) and phosphorylation-mimic (CtSD) PAPC were able to pull-down β-TrCP. Non-phosphorylated form of PAPC C-terminal domain (CtSA) did not interact with β-TrCP. (**C**) The ubiquitination assay showed that PAPC is poly-ubiquitinated in *Xenopus* embryos but that this was abolished by expressing dominant-negative β-TrCP (β-TrCPΔF). (**D**) Non-phosphorylated form of PAPC (PAPC-SA13) and PAPC with lysine-to-arginine mutations in the cytoplasmic region (PAPC-KR) exhibited markedly reduced poly-ubiquitination. (**E**) The amino acid sequences of the PAPC DSR domain and the β-catenin destruction motif (DM) are weakly similar. The localizations of GFP-tagged PAPCΔDSR and GFP-tagged PAPC-β-catDM (PAPC whose DSR domain was replaced by β-catenin DM) were shown. The graph shows the relative signal intensity in the cytoplasm (c) and on the plasma membrane (m).

We then examined the physical interaction between PAPC and β-TrCP in vitro by GST pull-down assays. Here, all the proteins were expressed in *Xenopus* embryos and purified for the analyses. Myc-tagged β-TrCP (Myc-TrCP) was pulled-down with GST-tagged PAPC (PAPC-GST), and this was abolished by deleting the substrate recognition domain (WD40 domain) of β-TrCP (Myc-TrCPΔWD40) (Fig. 7A). The cytoplasmic domain of PAPC tagged with GST (GST-Ct) was also able to pull-down Myc-TrCP (Fig. 7A). Introducing mutations equivalent to ‘phosphorylation-mimic’ PAPC-SD to GST-Ct (GST-Ct-SD) did not alter its binding to β-TrCP (Fig. 7B), while mutations equivalent to ‘non-phosphorylated’ mutant PAPC-SA (GST-Ct-SA) abrogated its interaction with β-TrCP (Fig. 7B), indicating that phosphorylation at the DRS domain may be required for the PAPC-β-TrCP interaction. The ubiquitination assay further revealed that PAPC is ubiquitinated in *Xenopus* cells in a β-TrCP-dependent manner, as expression of TrCPΔF suppressed PAPC ubiquitination observed in the control (Fig. 7C). As expected, ubiquitination was greatly reduced in the PACP-KR mutant (Fig. 7D and S3 Fig.). Furthermore, the ‘non-phosphorylated’ mutation (PAPC-SA-GST) also attenuated PAPC ubiquitination (Fig. 7D), and this coincided with the lack of interaction between GST-Ct-SA and Myc-TrCP.

β-catenin is phosphorylated by GSK3 and ubiquitinated by β-TrCP. The PAPC DSR sequence shows weak similarity to the destruction motif (DM) [18], which contains a GSK3 phosphorylation site in β-catenin (Fig. 7E). We thus examined whether the β-catenin DM could functionally replace PAPC DSR. The PAPC mutant whose DSR region was deleted (PAPCΔDSR) formed large aggregates (Fig. 7E). When the β-catenin DM sequence was inserted into the same site as the DSR region (PAPC-β-catDM), its localization was more similar to wild-type PAPC (Figs. 7E and 1A). This result indicates that β-catenin DM can replace the DSR domain. Together, it is strongly suggested that phosphorylation of PAPC by GSK3 in the DSR domain is required for the PAPC-β-TrCP interaction and hence PAPC ubiquitination by β-TrCP, and that this system is important for the regulation of PAPC localization and stability.

## Discussion

In this study we demonstrated that PAPC is down-regulated in the axial mesoderm during gastrulation, not only at the transcriptional level but also at the protein level, by ubiquitin-mediated degradation. Our results show that phosphorylation of PAPC by GSK3 serves as a signal for ubiquitination of PAPC by β-TrCP, and this process is necessary for PAPC to enter the normal regulation cycle. At an early gastrula stage (stage 11), PAPC is recycled to the plasma membrane, while by the mid-neurula stage (stage 15) PAPC is largely degraded and excluded from the plasma membrane in the axial mesoderm. The dual-regulation system at both transcriptional and protein levels may facilitate the rapid change of PAPC levels within the tissue.

The early presence and later absence of PAPC in the axial mesoderm during gastrulation seem to be crucial for *Xenopus* embryogenesis, as both MO knock-down and mRNA over-expression of PAPC lead to deficient gastrulation cell movements. Here, PAPC may have cell-autonomous and non-cell-autonomous functions. Unterseher et al. [5] showed that in the vegetal alignment zone, PAPC is cell-autonomously required for co-ordination of cell polarity at an early gastrula stage. Our data suggest that later on in gastrulation, the persistence of PAPC on the plasma membrane may inhibit cell motility and CE cell movement in this region. Given that the chordamesoderm is a highly cohesive tissue in terms of surface tension and cadherin expression, it is reasonable that these cells have to reduce cell-cell adhesion to facilitate intercalation movement. The inhibition of elongation of mesodermalized animal caps by PAPC over-expression also supports the idea of a cell-autonomous function. Additionally, PAPC expression in the paraxial mesoderm may exert a non-cell-autonomous effect on the axial mesoderm. Unterseher et al. [5] observed that PAPC inhibition caused CE defects in the axial mesoderm, but not in the paraxial mesoderm where PAPC is expressed. PAPC is implicated in a variety of molecular networks, and its nature in cell adhesion and signalling has to be clarified to unravel its precise role in cell movements.

We demonstrated that the highly conserved region, which we designated as the ‘DSR domain’, plays a key role in the regulation of PAPC localization and stability. We focused on the conserved serine residues in the domain. The mutant whose DSR domain was deleted formed large cytoplasmic aggregates. These aggregates are stable in *Xenopus* mesoderm cells until later developmental stages (Fig. 1A). Similar clustering was observed in PAPC-SA1, which has mutations in the conserved serine residues in the DSR domain (Fig. 2B). These aggregates seemed to be clusters of endosomal vesicles because (i) they were co-localized with an early endosomal marker, EEA1 (Fig. 3A and B), and (ii) the cluster formation was inhibited by suppressing endocytosis with the dominant-negative dynamin [10], although as the results were obtained through over-expression studies the possibility of formation of aggresome-like structure cannot be totally excluded. Nonetheless, our data demonstrated that the DSR domain plays an important role in the regulation of PAPC localization and stability.

We found that phosphorylation by GSK3 and ubiquitination by β-TrCP are involved in this regulatory PAPC system. This is expected since the DSR domain is similar to the amino acid sequence around the GSK3 phosphorylation sites of β-catenin (Fig. 7E). The phosphorylation of β-catenin triggers ubiquitination by β-TrCP E3 ligase. PAPC-SA1 has a mutation in the SXXXS motif, which is a known potential target sequence for GSK3 [19] DN-GSK3 and dominant-negative β-TrCP (TrCPΔF) expression induced PAPC clustering similar to the PAPC-SA1 mutant, suggesting the role of GSK3 and β-TrCP in PAPC regulation. Furthermore, we demonstrated that PAPC is phosphorylated and ubiquitinated in *Xenopus* embryos. We found that the destruction box of β-catenin can functionally replace the DSR domain of PAPC in terms of PAPC localization in animal cap cells (Fig. 7E). This indicates that the regulation of protein localization and/or stability via phosphorylation, ubiquitination and deubiquitination through DSR-like sequences may be used in diverse cellular processes. PAPC belongs to the δ2-protocadherin family, whose members include PCDH8, PCDH10 and PCDH18 [20]. The DSR-like sequence is conserved among several protocadherins, particularly in serine residues. We found that mouse PCDH10 [21] expressed in *Xenopus* DMZ cells is down-regulated during the neurula stage and co-localized with β-TrCP as well as PAPC (S4 Fig.), suggesting that PCDH10 and other family members are also regulated by a similar mechanism.

Because GSK3 and β-TrCP are components of the canonical Wnt pathway, it is intriguing that the Wnt pathway may regulate PAPC localization and stability. Kraft et al. (2012) reported that PAPC localization is regulated by the Wnt/PCP pathway [10]. Inhibition of Wnt/PCP signalling induces PAPC internalization while Xwnt11 expression increases the plasma membrane localization of PAPC. The canonical and non-canonical Wnt pathways are known to antagonize each other [22]. It is also of interest to note that the canonical Wnt pathway represses notochord formation [23] [24], while promoting paraxial mesoderm fate [25] [26]. Considering these data, the canonical Wnt pathway may also be involved in the PAPC regulatory system by controlling GSK3 and β-TrCP activity.

What is the role of phosphorylation and ubiquitination in the DSR domain? The inhibition of these reactions maintains PAPC in the cytoplasmic endosomes, suggesting that these reactions might promote the recycling of PAPC back to the plasma membrane or promote PAPC degradation. We detected PAPC-GFP on the plasma membrane of the *Xenopus* mesoderm during early gastulation, but it was almost undetectable at a later stage when convergent extension movement occurs. Thus, we hypothesize that ubiquitination promotes PAPC recycling in an early stage, and degradation in a later stage. This distinction is to be determined in future experiments. It has been reported that mono-ubiquitination or lysine-63 linked poly-ubiquitination leads to internalization of many membrane proteins [27]. In the case of PAPC, ubiquitination does not seem to be a trigger for endocytosis. Since β-TrCP E3 ligase forms lysine-48 linked poly-ubiquitin, PAPC is also expected to be ubiquitinated in the same way. Thus, this regulatory system must be a novel mechanism to regulate membrane protein localization and stability.

A question remains how PAPC is differentially controlled at different developmental time and space, such that it is recycled to the plasma membrane at stage 11 while degraded specifically in the axial mesoderm by stage 15. Ubiquitinated proteins are regulated at different levels by their associated proteins. One such protein, valosin-containing protein (VCP)/p97, can structurally remodel ubiquitinated clients as well as edit ubiquitin modification, and helps process ubiquitin-labelled proteins for recycling or degradation [28]. There are also a large number of deubiquitinase (DUB) species that act to remove ubiquitin from the client proteins. These modifiers of ubiquitin may be regulated temporally and spatially to determine the fate of PAPC.

In conclusion, we demonstrated that phosphorylation of PAPC by GSK3 and subsequent poly-ubiquitination by β-TrCP is crucial for regulation of PAPC localization and stability. At the early gastrula stage, PAPC is recycled to the plasma membrane, while by the early neurula stage, PAPC is degraded and excluded from the plasma membrane in the axial mesoderm. Disruption of this system in *Xenopus* early embryos leads to abnormal morphogenesis typical of gastrulation defects. Our findings suggest a novel mechanism for tuning cell-cell adhesion levels by the ubiquitin system, which plays a crucial role in vertebrate embryogenesis.

## Materials and Methods

### *Xenopus* embryo manipulations

*Xenopus* eggs were obtained from females injected with 400 IU of human chorionic gonadotrophin (Takeda Pharm., Japan), and were fertilized in vitro. Eggs were dejellied with 3% cysteine hydrochloride (pH 7.8) and embryos were microinjected in 0.1× Steinberg’s solution [29] containing 3% Ficoll400. The embryos were staged according to Nieuwkoop and Faber [30].

This study was carried out in strict accordance with the Guide for the Care and Use of Laboratory Animals of National Institute for Basic Biology. The protocol was approved by the Institutional Animal Care and Use Committee of National Institutes of Natural Sciences (Permit Number: 12A002).

### Plasmid construction, Morpholono oligonucleotides and injection

The cDNAs encoding *Xenopus laevis* PAPC, β-TrCP and GSK3 were obtained from our XDB *Xenopus* EST collection (http://xenopus.nibb.ac.jp). The ubiquitin constructs are described previously [31] [32]. All the cDNA fragments were cloned into pCS2+ vector. Deletion and missense mutations were introduced by PCR-based mutagenesis. PAPCΔC lacks the cytoplasmic domain of PAPC that encodes the 1–740 amino-acid region. The dominant-negative dynamin construction (K46A) is described in [31].

The PAPC MOs (Gene Tools LLC, Philomath, USA) were described previously [5]. The β-TrCP Morpholino oligonucleotide sequence was 5′-GAGAACATGAAAATCCTTCCATCTC-3′ while that of the control MO was 5′-CCTCTTACCTCAGTTACAATTTATA-3′. 10 pmol of MOs was injected into one embryo.

mRNAs were synthesized using mMESSAGE mMACHINE SP6 kit (Ambion, Austin, TX) following the manufacturer’s instruction. The amounts of mRNA for injection were 500 pg for PAPC and its mutant forms unless otherwise stated, and 100 pg for membrane RFP, ubiquitin, β-TrCPΔF, EEA1 and DN-dynamin.

GSK3 inhibitor BIO (Sigma, St Louis, MO) was dissolved in DMSO at 20 mM. For treatment of BIO, the vitelline membrane was removed at stage 10.5 and embryos were soaked in 1× Steinberg’s solution containing 20 μM for one hour. Then, explants were dissected and observed by microscope.

### RT-PCR analysis

The procedure for RT-PCR analysis was described previously [33]. Briefly, RNA from *Xenopus* embryos and explants was prepared with Trizol (Life Technologies). cDNA was synthesized with Reverse Transcriptase (TRT-101, Toyobo, Japan). Sequences of the primers for *chordin, goosecoid*, and *ODC* were as described on Dr. De Robertis’ home page (http://www.hhmi.ucla.edu/derobertis/index.html).

### Microscopic observation

A detailed procedure for microscopic observations is described in Iioka et al. [34]. In brief, *Xenopus* embryonic explants were dissected and incubated in 1× Steinberg’s solution. For microscopic observation, explants were placed on a glass bottom dish and observed by laser-scanning confocal microscopy (Zeiss LSM510 Meta). Fluorescent images were analysed using ImageJ software (http://imagej.nih.gov/ij/).

### Antibodies

Mouse monoclonal antibodies against GST and Myc-epitope tag were purchased from Santa Cruz (cat # sc-138 and sc-10, respectively). Polyclonal antibodies against phosphorylated PAPC were raised by immunizing rabbits with a synthetic peptide ‘MGHI(pS)TKD(pS)GKGD’. ‘(pS)’ indicates a phosphorylated serine residue. The antisera were affinity-purified with the phosphorylated peptide, followed by absorption of the non-phosphorylated peptide ‘MGHISTKDSGKGD’.

### *In vivo* phosphorylation assay

PAPC-GST mRNA (500 pg) was injected into the dorsal blastomeres of four-cell embryos. When the embryos reached stage 11, they were squashed by pipetting in PBS containing 0.3% Triton X-100, protease inhibitor and phosphatase inhibitor cocktail (Nacalai Tesque, Kyoto, Japan). The homogenates were centrifuged for 10 min at 14,000 rpm, and then the supernatants were mixed with 15 μl of Glutathione Sepharose 4B beads (GE Healthcare). The tubes were rotated with a tumbling mixer. After 60 min, the beads were washed three times with the same buffer. For the phosphatase treatment, beads were treated with or without 400 units of lambda protein phosphatase (#P0753, New England Biolabs) at 30°C for 60 min. The beads were washed once in PBS 0.3% Triton X-100, and proteins bound to the beads were run in SDS-PAGE.

### *In vitro* phosphorylation assay

The cytoplasmic domain of PAPC (714–877 aa) fused to GST (PAPC Cterm-GST) was ligated into a bacterial expression vector pColdII (Takara, Shiga, Japan). The fusion protein was expressed in *E. coli* strain BL21(DE3) pLysS and purified with Glutathione Sepharose 4B. 10 μg of the protein was incubated with glycogen synthase kinase 3 (#P6040, New England Biolabs) at 30°C for 1 h according to the manufacturer’s instruction.

### *In vivo* ubiquitination assay

mRNAs encoding PAPC-GST (500 pg) and myc-ubiquitin (100 pg) were co-injected into the dorsal blastomeres of four-cell embryos. When the embryos reached stage 11, 20 embryos were squashed by pipetting in 500 μl of PBS containing 0.3% Triton X-100 and protease inhibitor cocktail (Nacalai Tesque). The homogenates were centrifuged for 10 min at 14,000 rpm, and the supernatants were mixed with 15 μl of Glutathione Sepharose 4B beads. The tubes were rotated with a tumbling mixer. After 60 min, the beads were washed three times with the same buffer and proteins bound to the beads were analyzed by Western blotting.

## Supporting Information

S1 Fig PAPC stability in the notochord. (**A**) EGFP-PAPCDE construction. The C-terminally tagged PAPC-GFP over-expression impairs gastrulation, probably due to its activity as a cell adhesion molecule (see Fig. 1). We thus constructed EGFP-PAPCΔE, in which the ectodomain of PAPC is replaced with EGFP. The C-terminal part of PAPC (678–979 aa), containing the transmembrane domain (tm) and cytoplasmic domain, was fused to EGFP. The signal sequence (ss) was derived from *Xenopus* C-cadherin (1–31 aa). EGFP-PAPCΔE expression did not affect the notochord formation as shown below. (B and C) EGFP-PAPCΔE and membrane-RFP mRNAs were injected into the dorsal blastomeres of four-cell embryos. (**B**) At the onset of gastrulation (stage 10.5), dorsal marginal zone explants were dissected and cultured on a fibronectin-coated dish until the sibling embryos reached stage 17. The dotted line indicates the notochord boundary. (**C**) The embryos were fixed at stage 17 and cross-sections were made (200 μm thickness). The dotted circle indicates the notochord. BF; bright field. EGFP-PAPCΔE protein levels were quantified by measuring fluorescent intensities (B and C).
(TIF)

S2 Fig (A) PAPC-SA1 localizes to the cytoplasmic vesicles in a dose-independent manner. 500 pg (upper panels) and 100 pg (lower panels) of PAPC-SA1 mRNA was injected with membrane-RFP mRNA. Even at the lower dose, most of PAPC-SA1 localized in the cytoplasmic vesicles. Arrow heads indicate some of small and faint vesicles. (B) Protein levels of PAPC-GFP (WT) and PAPC-SA1-GFP (SA1). mRNAs encoding these proteins were expressed with membrane-RFP in *Xenopus* embryos. At stage 10.5, embryonic lysates were prepared and Western blotting was done.
(TIF)

S3 Fig Ubiquitination was detected with highly-purified PAPC. Different PAPC constructs indicated by the numbers (1–3) were expressed in *Xenopus* embryos. The constructs were doubly-tagged by GST and 6×-His, separated by the TEV protease recognition sequence. Purification was performed in a multi-step procedure, firstly with GST pull-down, and secondly, after elution by TEV protease (Sigma) digestion, with Ni-NTA agarose beads (Qiagen). Ni-agarose precipitation was done in 6 M urea, in which most proteins were supposed to be denatured. Even in this condition, Myc-Ubi ladder was detected with wild-type PAPC, strongly suggesting that ubiquitins were directly and covalently bound to PAPC.
(TIF)

S4 Fig Mouse PCDH10 localization. Mouse PCDH10 (mPCDH10) is regulated in the way similar to PAPC. (**A**) GFP-tagged mPCDH10 and membrane-RFP (mRFP) were expressed in a DMZ explant. mPCDH10 was expressed at stage 11 (gastrula) but donw-regulated at stage 17 (neurula). (**B**) GFP-tagged mPCDH10 and RFP-tagged β-TrCP were expressed in a DMZ explant. They were co-localized particularly in the cytoplasmic vesicles.
(TIF)

S5 Fig Unprocessed gel and Western data (1). Images which were cut and used in the main figures are indicated by boxes. Lanes not used for the main figures were not related to this work.
(TIF)

S6 Fig Unprocessed gel and Western data (2). Images which were cut and used in the main Figures are indicated by boxes. Lanes not used for the main figures were not related to this work.
(TIF)

## References

[1] Keller R (2002) Shaping the vertebrate body plan by polarized embryonic cell movements. Science 298: 1950–1954. 10.1126/science.1079478 12471247

[2] Winklbauer R (2009) Cell adhesion in amphibian gastrulation. Int Rev Cell Mol Biol 278: 215–275. 10.1016/S1937-6448(09)78005-0 19815180

[3] Kim SH, Yamamoto A, Bouwmeester T, Agius E, Robertis EM (1998) The role of paraxial protocadherin in selective adhesion and cell movements of the mesoderm during Xenopus gastrulation. Development 125: 4681–4690. 9806917 10.1242/dev.125.23.4681

[4] Yamamoto A, Amacher SL, Kim SH, Geissert D, Kimmel CB, et al. (1998) Zebrafish paraxial protocadherin is a downstream target of spadetail involved in morphogenesis of gastrula mesoderm. Development 125: 3389–3397. 9693142 10.1242/dev.125.17.3389 PMC2280034

[5] Unterseher F, Hefele JA, Giehl K, De Robertis EM, Wedlich D, et al. (2004) Paraxial protocadherin coordinates cell polarity during convergent extension via Rho A and JNK. EMBO J 23: 3259–3269. 10.1038/sj.emboj.7600332 15297873 PMC514506

[6] Wang Y, Janicki P, Koster I, Berger CD, Wenzl C, et al. (2008) Xenopus Paraxial Protocadherin regulates morphogenesis by antagonizing Sprouty. Genes Dev 22: 878–883. 10.1101/gad.452908 18381892 PMC2279199

[7] Kim SH, Jen WC, De Robertis EM, Kintner C (2000) The protocadherin PAPC establishes segmental boundaries during somitogenesis in xenopus embryos. Curr Biol 10: 821–830. 10.1016/S0960-9822(00)00580-7 10899001

[8] Medina A, Swain RK, Kuerner KM, Steinbeisser H (2004) Xenopus paraxial protocadherin has signaling functions and is involved in tissue separation. EMBO J 23: 3249–3258. 10.1038/sj.emboj.7600329 15272309 PMC514504

[9] Chen X, Gumbiner BM (2006) Paraxial protocadherin mediates cell sorting and tissue morphogenesis by regulating C-cadherin adhesion activity. J Cell Biol 174: 301–313. 10.1083/jcb.200602062 16847104 PMC2064189

[10] Kraft B, Berger CD, Wallkamm V, Steinbeisser H, Wedlich D (2012) Wnt-11 and Fz7 reduce cell adhesion in convergent extension by sequestration of PAPC and C-cadherin. J Cell Biol 198: 695–709. 10.1083/jcb.201110076 22908314 PMC3514027

[11] Rhee J, Takahashi Y, Saga Y, Wilson-Rawls J, Rawls A (2003) The protocadherin papc is involved in the organization of the epithelium along the segmental border during mouse somitogenesis. Dev Biol 254: 248–261. 10.1016/S0012-1606(02)00085-4 12591245

[12] Chung HA, Yamamoto TS, Ueno N (2007) ANR5, an FGF target gene product, regulates gastrulation in Xenopus. Curr Biol 17: 932–939. 10.1016/j.cub.2007.04.034 17475493

[13] Komander D, Rape M (2012) The ubiquitin code. Annu Rev Biochem 81: 203–229. 10.1146/annurev-biochem-060310-170328 22524316

[14] Mukhopadhyay D, Riezman H (2007) Proteasome-independent functions of ubiquitin in endocytosis and signaling. Science 315: 201–205. 10.1126/science.1127085 17218518

[15] Chen X, Molino C, Liu L, Gumbiner BM (2007) Structural elements necessary for oligomerization, trafficking, and cell sorting function of paraxial protocadherin. J Biol Chem 282: 32128–32137. 10.1074/jbc.M705337200 17823115

[16] Pierce SB, Kimelman D (1995) Regulation of Spemann organizer formation by the intracellular kinase Xgsk-3. Development 121: 755–765. 7720580 10.1242/dev.121.3.755

[17] Nakayama KI, Nakayama K (2005) Regulation of the cell cycle by SCF-type ubiquitin ligases. Semin Cell Dev Biol 16: 323–333. 10.1016/j.semcdb.2005.02.010 15840441

[18] Liu C, Kato Y, Zhang Z, Do VM, Yankner BA, et al. (1999) beta-Trcp couples beta-catenin phosphorylation-degradation and regulates Xenopus axis formation. Proc Natl Acad Sci U S A 96: 6273–6278. 10.1073/pnas.96.11.6273 10339577 PMC26871

[19] Bax B, Carter PS, Lewis C, Guy AR, Bridges A, et al. (2001) The structure of phosphorylated GSK-3beta complexed with a peptide, FRATtide, that inhibits beta-catenin phosphorylation. Structure 9: 1143–1152. 10.1016/S0969-2126(01)00679-7 11738041

[20] Redies C, Vanhalst K, Roy F (2005) delta-Protocadherins: unique structures and functions. Cell Mol Life Sci 62: 2840–2852. 10.1007/s00018-005-5320-z 16261259 PMC11138374

[21] Uemura M, Nakao S, Suzuki ST, Takeichi M, Hirano S (2007) OL-Protocadherin is essential for growth of striatal axons and thalamocortical projections. Nat Neurosci 10: 1151–1159. 10.1038/nn1960 17721516

[22] Weidinger G, Moon RT (2003) When Wnts antagonize Wnts. J Cell Biol 162: 753–755. 10.1083/jcb.200307181 12952929 PMC2172824

[23] Christian JL, Moon RT (1993) Interactions between Xwnt-8 and Spemann organizer signaling pathways generate dorsoventral pattern in the embryonic mesoderm of Xenopus. Genes Dev 7: 13–28. 10.1101/gad.7.1.13 8422982

[24] Hoppler S, Brown JD, Moon RT (1996) Expression of a dominant-negative Wnt blocks induction of MyoD in Xenopus embryos. Genes Dev 10: 2805–2817. 10.1101/gad.10.21.2805 8946920

[25] Martin BL, Kimelman D (2012) Canonical Wnt signaling dynamically controls multiple stem cell fate decisions during vertebrate body formation. Dev Cell 22: 223–232. 10.1016/j.devcel.2011.11.001 22264734 PMC3465166

[26] Nowotschin S, Ferrer-Vaquer A, Concepcion D, Papaioannou VE, Hadjantonakis AK (2012) Interaction of Wnt3a, Msgn1 and Tbx6 in neural versus paraxial mesoderm lineage commitment and paraxial mesoderm differentiation in the mouse embryo. Dev Biol 367: 1–14. 10.1016/j.ydbio.2012.04.012 22546692 PMC3367124

[27] Haglund K, Dikic I (2005) Ubiquitylation and cell signaling. EMBO J 24: 3353–3359. 10.1038/sj.emboj.7600808 16148945 PMC1276169

[28] Meyer H, Bug M, Bremer S (2012) Emerging functions of the VCP/p97 AAA-ATPase in the ubiquitin system. Nat Cell Biol 14: 117–123. 10.1038/ncb2407 22298039

[29] Peng HB (1991) Xenopus laevis: Practical uses in cell and molecular biology. Solutions and protocols. Methods Cell Biol 36: 657–662. 1811156

[30] Nieuwkoop PD, Faber J (1975) Normal Table of Xenopus laevis (Daudin). Amsterdam, North Holland.

[31] Lee RH, Iioka H, Ohashi M, Iemura S, Natsume T, et al. (2007) XRab40 and XCullin5 form a ubiquitin ligase complex essential for the noncanonical Wnt pathway. EMBO J 26: 3592–3606. 10.1038/sj.emboj.7601781 17627283 PMC1949004

[32] Iioka H, Iemura S, Natsume T, Kinoshita N (2007) Wnt signalling regulates paxillin ubiquitination essential for mesodermal cell motility. Nat Cell Biol 9: 813–821. 10.1038/ncb1607 17558393

[33] Kinoshita N, Iioka H, Miyakoshi A, Ueno N (2003) PKC delta is essential for Dishevelled function in a noncanonical Wnt pathway that regulates Xenopus convergent extension movements. Genes Dev 17: 1663–1676. 10.1101/gad.1101303 12842914 PMC196137

[34] Iioka H, Ueno N, Kinoshita N (2004) Essential role of MARCKS in cortical actin dynamics during gastrulation movements. J Cell Biol 164: 169–174. 10.1083/jcb.200310027 14718521 PMC2172330

