# Supplementary figures and images for: Phosphorylation-Dependent Ubiquitination of Paraxial Protocadherin (PAPC) Controls Gastrulation Cell Movements
Source: PLoS One. 2015 Jan 12;10(1):e0115111. doi: 10.1371/journal.pone.0115111 (PMC4291225; doi:10.1371/journal.pone.0115111)

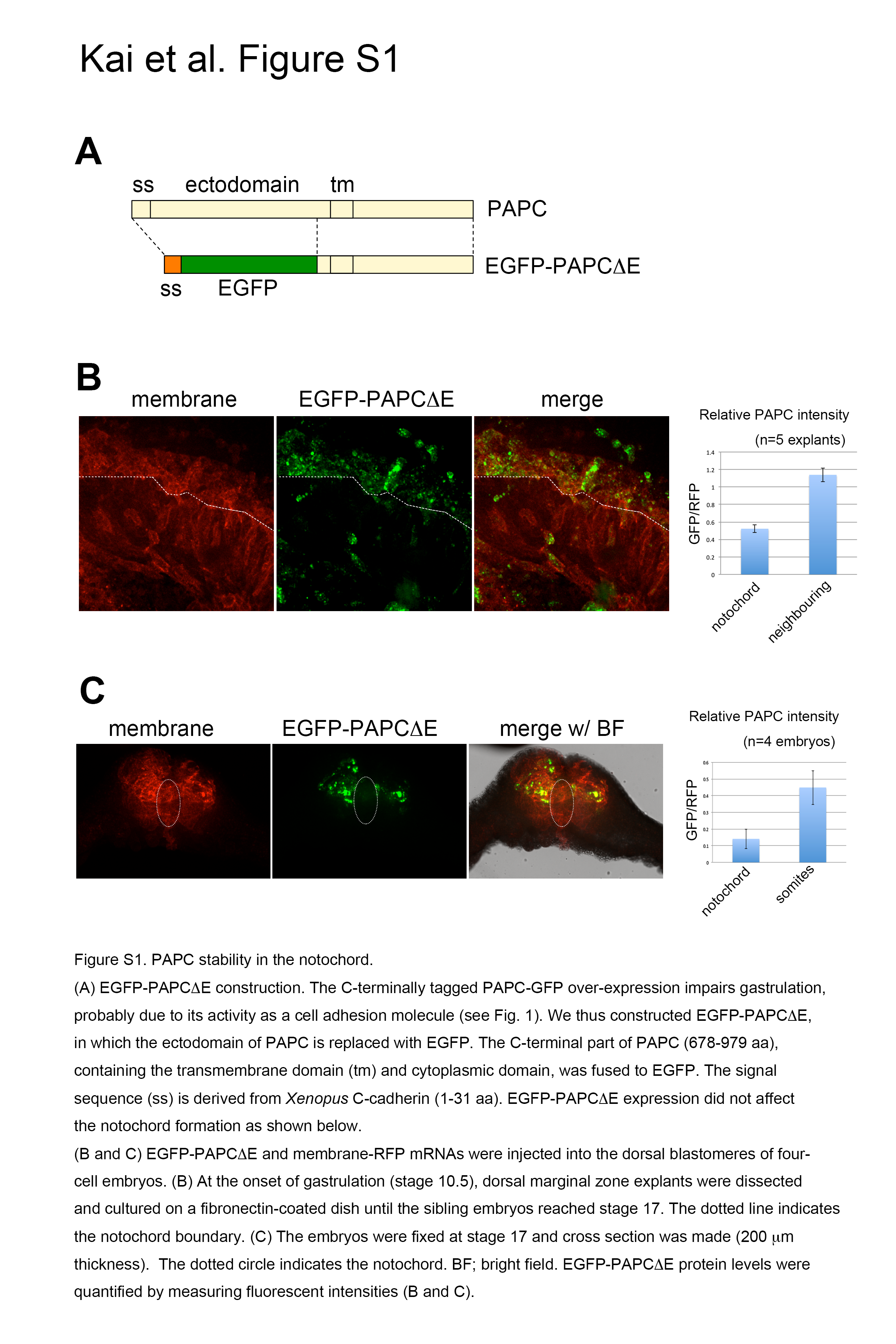

Supplement: S1 Fig — (A) EGFP-PAPCDE construction. The C-terminally tagged PAPC-GFP over-expression impairs gastrulation, probably due to its activity as a cell adhesion molecule (see Fig. 1). We thus constructed EGFP-PAPCΔE, in which the ectodomain of PAPC is replaced with EGFP. The C-terminal part of PAPC (678–979 aa), containing the transmembrane domain (tm) and cytoplasmic domain, was fused to EGFP. The signal sequence (ss) was derived from Xenopus C-cadherin (1–31 aa). EGFP-PAPCΔE expression did not affect the notochord formation as shown below. (B and C) EGFP-PAPCΔE and membrane-RFP mRNAs were injected into the dorsal blastomeres of four-cell embryos. (B) At the onset of gastrulation (stage 10.5), dorsal marginal zone explants were dissected and cultured on a fibronectin-coated dish until the sibling embryos reached stage 17. The dotted line indicates the notochord boundary. (C) The embryos were fixed at stage 17 and cross-sections were made (200 μm thickness). The dotted circle indicates the notochord. BF; bright field. EGFP-PAPCΔE protein levels were quantified by measuring fluorescent intensities (B and C). (TIF) [file pone.0115111.s001.tif]

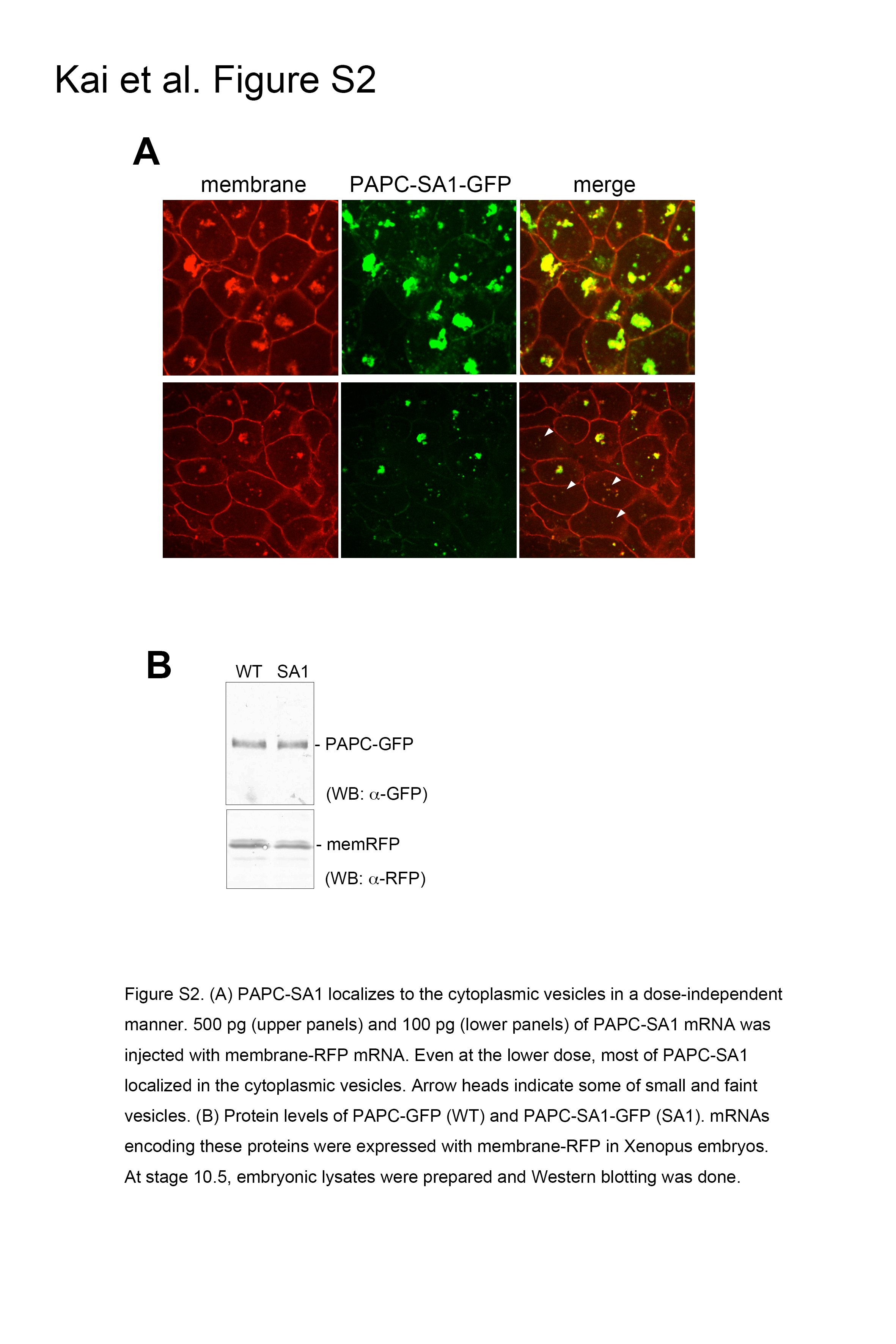

Supplement: S2 Fig — 500 pg (upper panels) and 100 pg (lower panels) of PAPC-SA1 mRNA was injected with membrane-RFP mRNA. Even at the lower dose, most of PAPC-SA1 localized in the cytoplasmic vesicles. Arrow heads indicate some of small and faint vesicles. (B) Protein levels of PAPC-GFP (WT) and PAPC-SA1-GFP (SA1). mRNAs encoding these proteins were expressed with membrane-RFP in Xenopus embryos. At stage 10.5, embryonic lysates were prepared and Western blotting was done. (TIF) [file pone.0115111.s002.tif]

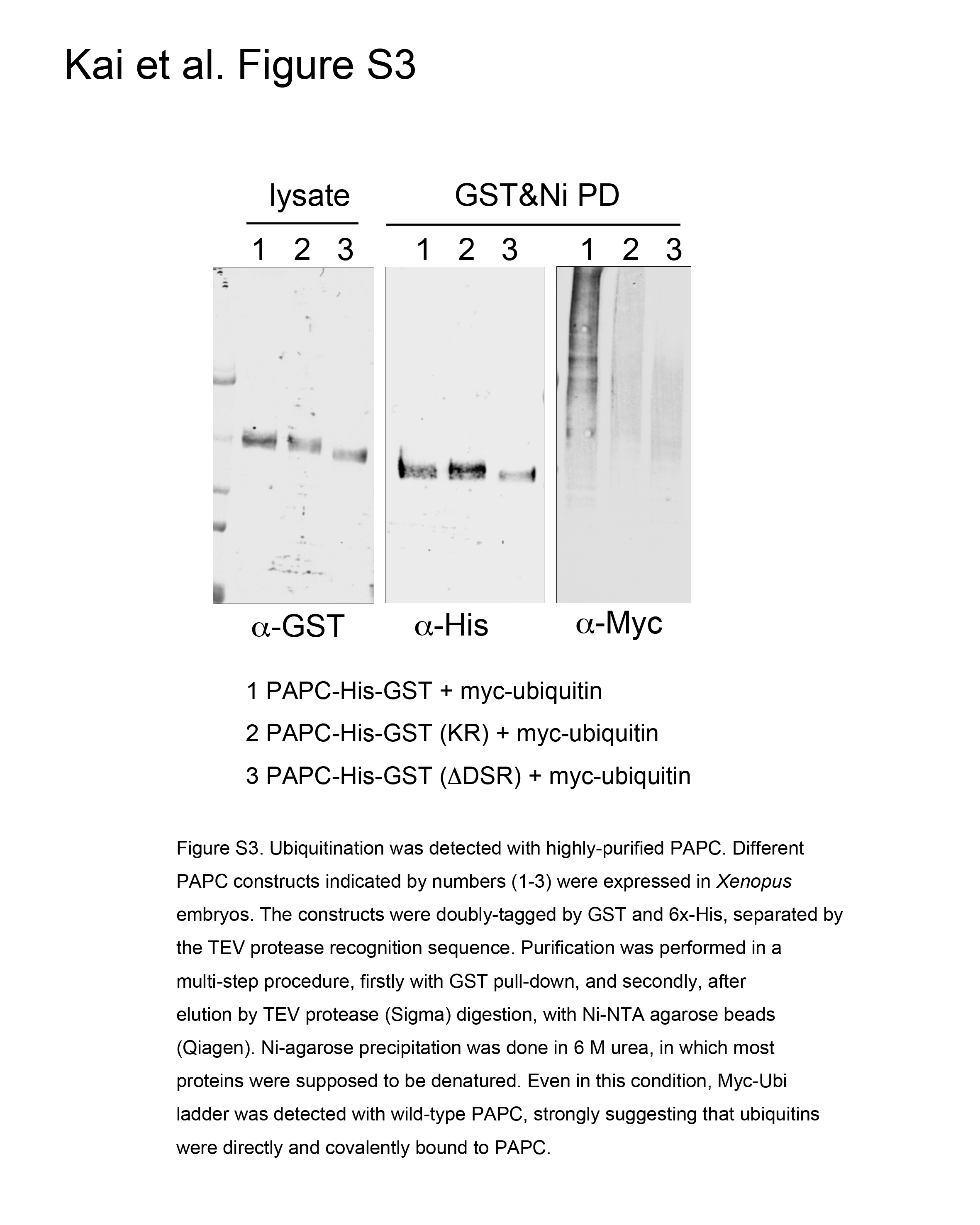

Supplement: S3 Fig — Different PAPC constructs indicated by the numbers (1–3) were expressed in Xenopus embryos. The constructs were doubly-tagged by GST and 6×-His, separated by the TEV protease recognition sequence. Purification was performed in a multi-step procedure, firstly with GST pull-down, and secondly, after elution by TEV protease (Sigma) digestion, with Ni-NTA agarose beads (Qiagen). Ni-agarose precipitation was done in 6 M urea, in which most proteins were supposed to be denatured. Even in this condition, Myc-Ubi ladder was detected with wild-type PAPC, strongly suggesting that ubiquitins were directly and covalently bound to PAPC. (TIF) [file pone.0115111.s003.tif]

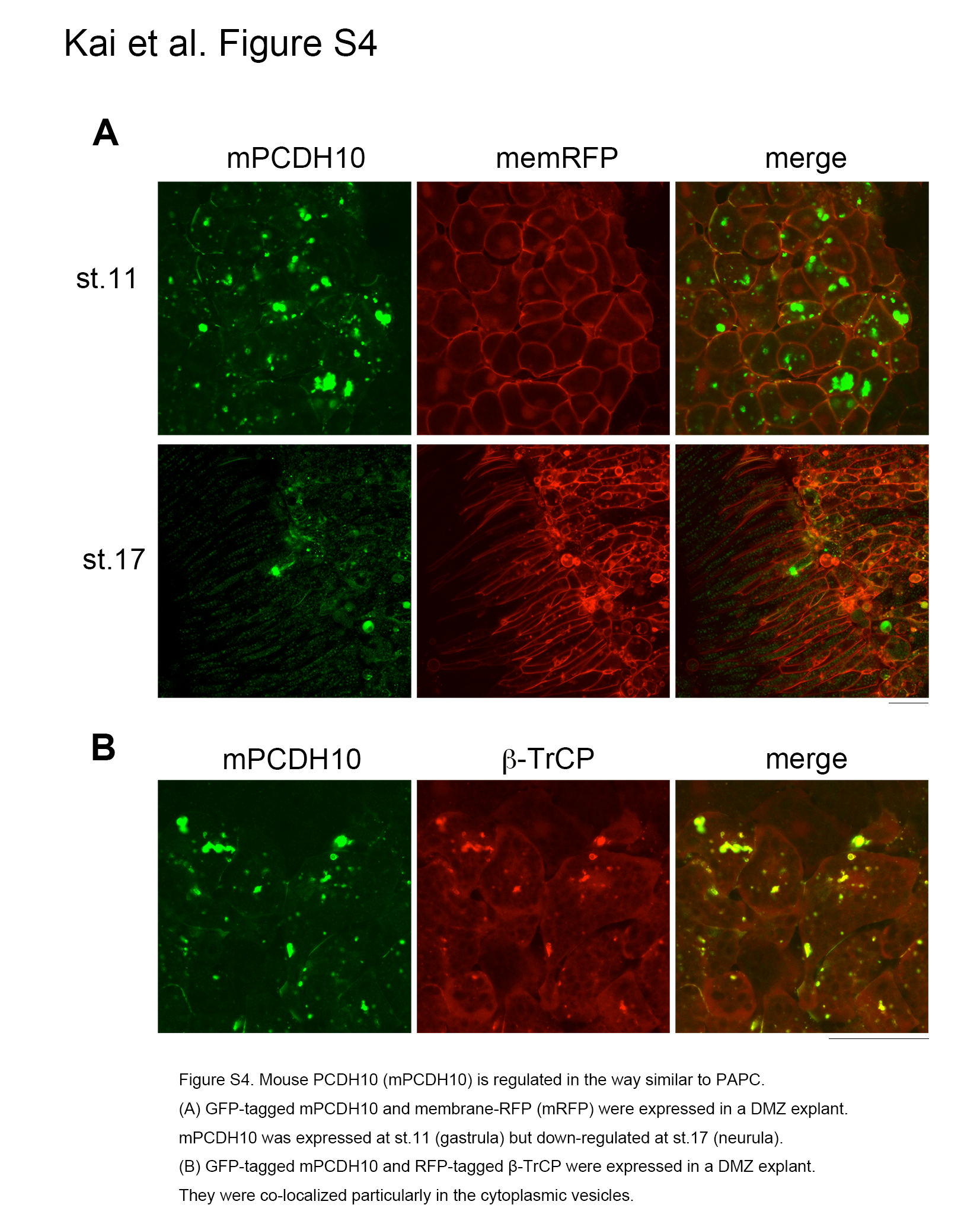

Supplement: S4 Fig — Mouse PCDH10 (mPCDH10) is regulated in the way similar to PAPC. (A) GFP-tagged mPCDH10 and membrane-RFP (mRFP) were expressed in a DMZ explant. mPCDH10 was expressed at stage 11 (gastrula) but donw-regulated at stage 17 (neurula). (B) GFP-tagged mPCDH10 and RFP-tagged β-TrCP were expressed in a DMZ explant. They were co-localized particularly in the cytoplasmic vesicles. (TIF) [file pone.0115111.s004.tif]

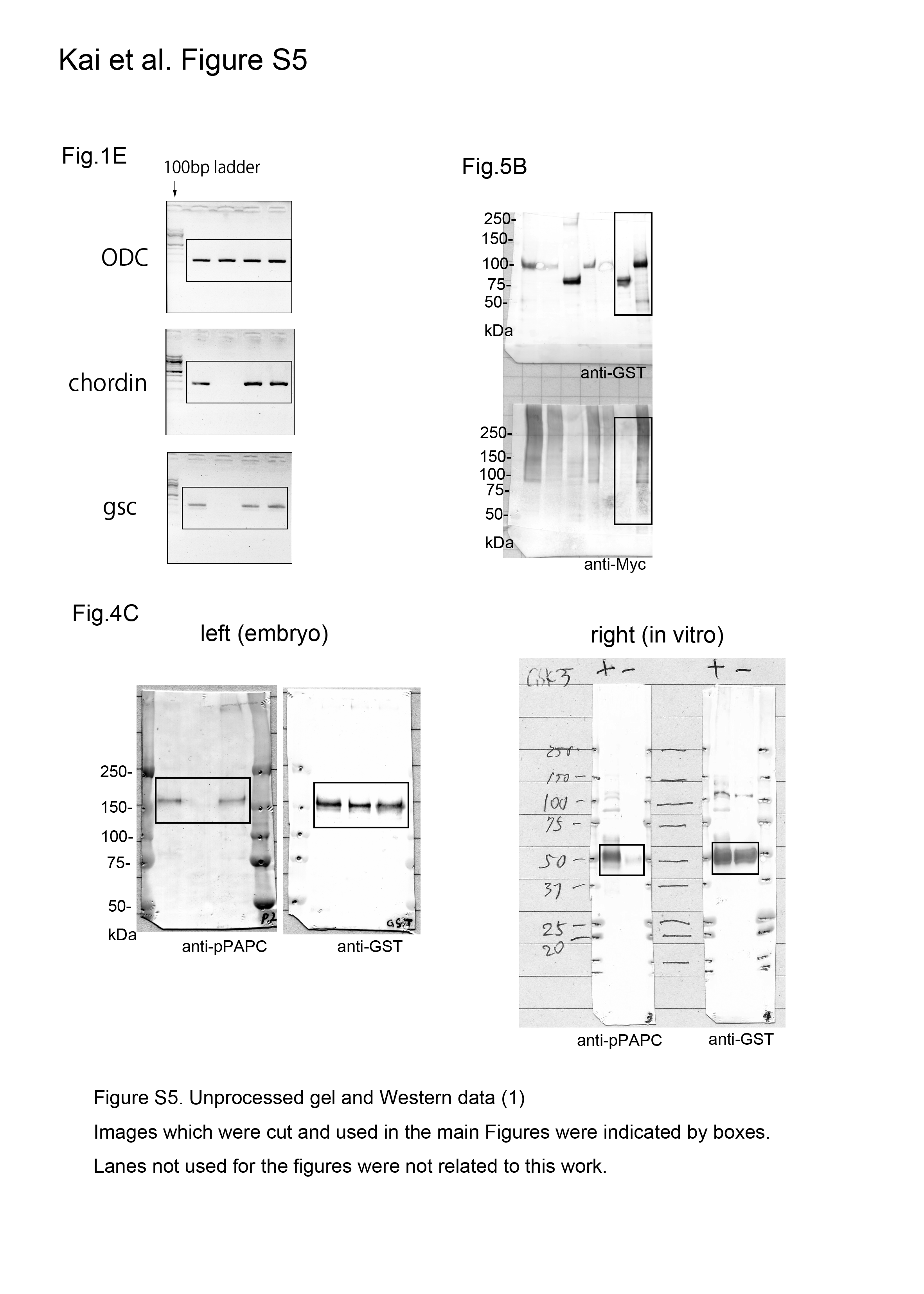

Supplement: S5 Fig — Images which were cut and used in the main figures are indicated by boxes. Lanes not used for the main figures were not related to this work. (TIF) [file pone.0115111.s005.tif]

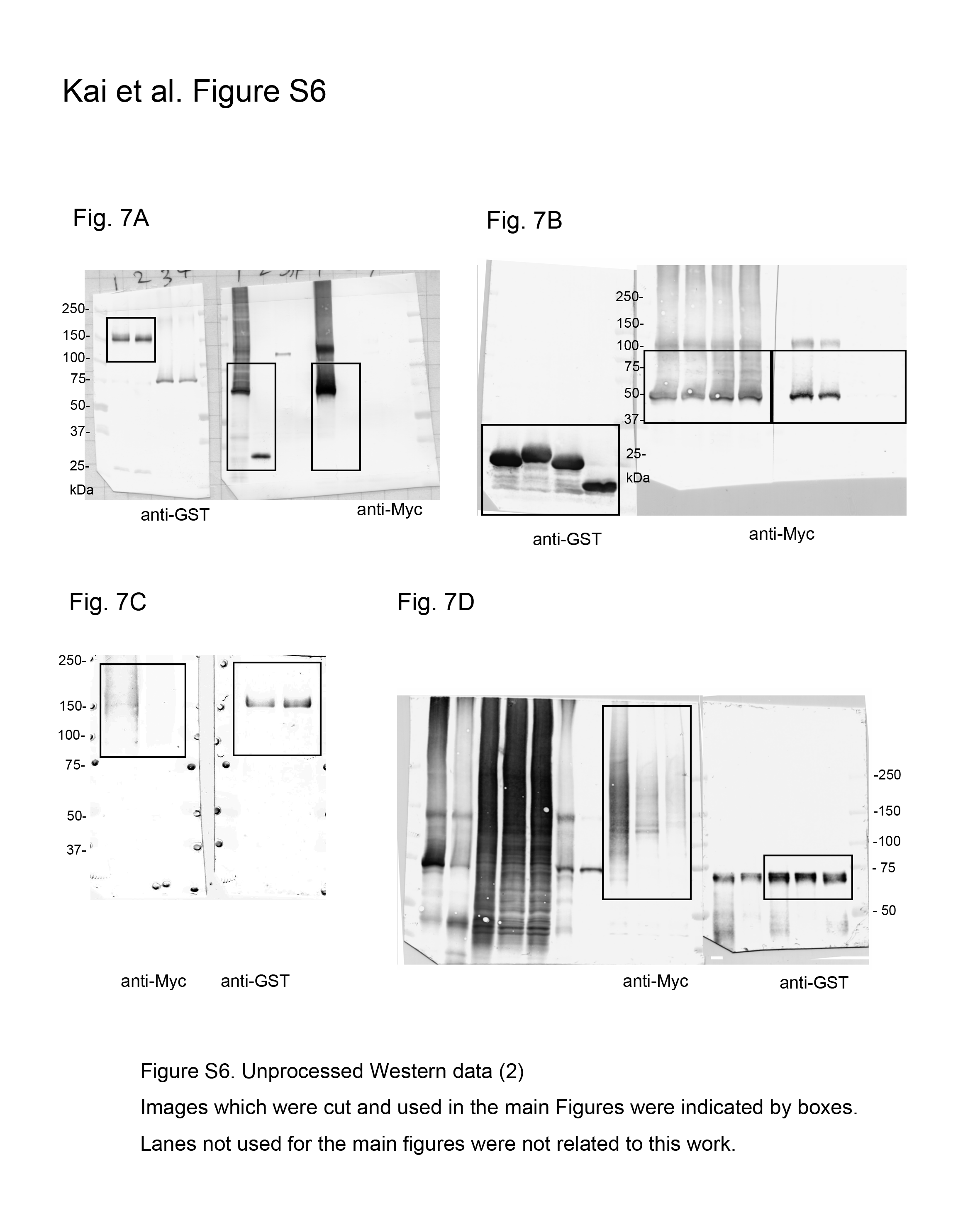

Supplement: S6 Fig — Images which were cut and used in the main Figures are indicated by boxes. Lanes not used for the main figures were not related to this work. (TIF) [file pone.0115111.s006.tif]
